# Exploring Information Access in Aging Populations and Those With Dementia and Mild Cognitive Impairment in the United Kingdom: Survey and Focus Group Study

**DOI:** 10.2196/85626

**Published:** 2026-04-21

**Authors:** Claire Rogers, William J McGeown, Yashar Moshfeghi

**Affiliations:** 1 NeuraSearch Laboratory, Department of Computer and Information Sciences, University of Strathclyde, 26 Richmond Street, Glasgow, United Kingdom, 44 1415483256; 2 Department of Psychological Sciences and Health, University of Strathclyde, Glasgow, United Kingdom

**Keywords:** artificial intelligence, dementia, mild cognitive impairment, older adult, generative AI, ChatGPT, search, information retrieval, aging, accessibility

## Abstract

**Background:**

With the growing aging population, technology that supports independent living is increasingly important. Web search systems are well established, whereas generative artificial intelligence (Gen-AI; eg, ChatGPT) represents a newer, adaptive tool that could offer personalized information access. However, little is known about how older adults, particularly those with mild cognitive impairment (MCI) or mild dementia, perceive and engage with these systems.

**Objective:**

This study explored the use of and perspectives on web search and Gen-AI in older adults with and without cognitive impairment (including MCI and early-stage dementia).

**Methods:**

A UK-wide mixed methods study was conducted with older adults, including those with MCI or mild dementia. An online survey captured technology use, Likert-scale ratings of web search and Gen-AI, and reasons for nonuse. Follow-up focus groups provided in-depth qualitative perspectives. Quantitative data were analyzed using descriptive and comparative statistics, while qualitative data were thematically analyzed.

**Results:**

Survey findings showed higher use of web search (275/280, 98.2%) compared to Gen-AI (40/286, 14%) within these groups. Web search was rated positively across participants, although challenges were raised regarding the phrasing of queries and commercialization. Gen-AI use was less common, but more than half of nonusers expressed willingness to adopt it in the future. Combined with focus group responses, themes exploring keyword searching, mistrust, lack of knowledge, and willingness to learn were established. Participants also suggested potential applications of Gen-AI, such as supporting independent living through monitoring and simplifying complex searches.

**Conclusions:**

Web search remains the primary method, and participants highlighted both advantages and frustrations with current systems. Gen-AI was underused but seen as promising, with its adoption mainly limited by mistrust and knowledge gaps. Our findings indicate that structured training, early introduction, and user-centered design could encourage adoption, enhance accessibility, and support independent living among older adults with and without MCI.

## Introduction

Given the rise in the number of people living with dementia worldwide [1], there is an increased demand for technologies that support independent living. In early-stage dementia and mild cognitive impairment (MCI), one potential technology to preserve independence is information retrieval (IR) systems that facilitate access to information. This includes 2 primary areas: web search (eg, Google) and generative artificial intelligence (Gen-AI), such as ChatGPT. In this study, “Gen-AI” refers to text-based conversational artificial intelligence (AI) systems powered by large language models (LLMs), such as ChatGPT. ChatGPT was used illustratively in the survey materials and focus groups, but perceptions of Gen-AI tools were examined more broadly. Much of the existing research on information access via web search has focused on users without impairments [2,3] or younger users [4-6], primarily examining health information seeking rather than broader applications [7-10]. Findings indicate that although health information seeking among older adults is becoming more common, barriers, including digital literacy, stigma, limited support, and information overload, remain [11,12]. Additionally, Gen-AI systems could serve as a promising tool for information access, enabling independent searches and conversations. Gen-AI’s natural language capabilities [13,14] suggest a more adaptable alternative to traditional web search [15], especially beneficial for those with limited digital skills or cognitive impairments.

Recent research suggests that Gen-AI could offer a more personalized and person-centered approach within both new and existing technologies [16], including information search, by simplifying complex information into more accessible formats [17,18]. This indicates that integrating such tools into information access mechanisms could be particularly advantageous in the context of aging [19]. Although many recognize the substantial ethical challenges posed by Gen-AI concerning data management and information sharing, especially given the vulnerability of this population [20,21], it is acknowledged that the potential of Gen-AI, particularly as a means of conversational search, remains significant [20]. Emerging studies have begun to explore older adults’ perspectives on LLM-based systems, especially regarding health information seeking and companionship [22], as well as wider AI-driven health technologies [19,23]. These studies suggest that while older adults see potential in these systems, they continue to face barriers such as limited digital literacy, ethical concerns, and persistent ageism in technology design. Yet, despite this growing body of research, little is known about how these tools are experienced by older adults with MCI or dementia, who may have different needs compared to their cognitively healthy peers. Addressing this gap is vital to ensure that future Gen-AI applications support, rather than exclude, those living with cognitive impairments. AI has also been identified as a potential means of enhancing quality of life through informational and social support [24], although studies have noted that responses from Gen-AI can lack the narrative richness and personal experiences often found in peer-to-peer online forums [25]. This highlights the need to balance efficiency with personalization and authenticity valued by older users. Research has begun to investigate their use in this cohort for IR [20], reducing loneliness [26-28], improving mental health [29], and establishing best training practices [30], indicating that the potential for application in the aging population has already been recognized.

Older adults and those with cognitive impairment are often stereotyped as unable or unwilling to use technology [31-33]. This may create a self-fulfilling prophecy [34-37] in which technology is perceived as irrelevant, leading to reluctance, avoidance [38,39], or an underestimation of their abilities. Unfortunately, this stereotype is reflected in some technological developments, which do not attempt to accommodate older populations or are overly simplistic, focusing on skills they have lost rather than retained [37,40]. Additionally, greater emphasis is often placed on young people and their use of technology, which can result in older adults being excluded from its benefits [41]. This “digital ageism” has become an increasingly recognized issue, highlighting how technologies can become misaligned with older adults as age-related changes occur, affecting digital literacy [42] and widening the divide as these groups remain underrepresented in technology development. Beyond equity concerns, reduced digital access has clinical implications, as it may limit autonomy, delay decision-making, or increase dependence on caregivers. More recently, researchers have stressed the importance of addressing this digital divide in relation to Gen-AI specifically, calling for greater attention to accessibility, user-centered design, and inclusivity [43]. Such work demonstrates that while Gen-AI could be transformative for older cohorts, deliberate efforts are necessary to realize these potential benefits. In recent years, the importance of capturing the views of underrepresented groups on AI systems has been increasingly recognized, with older adults identified as a key population whose perspectives should inform the development of these technologies [22]. Therefore, exploring perspectives on information access is vital to understanding how older adults with and without cognitive impairment (including MCI and early-stage dementia) use these technologies and where improvements are needed.

This paper examines usage patterns and perspectives of traditional IR and Gen-AI systems in healthy older adults, those with MCI, and those with early-stage dementia, to characterize current engagement and identify directions for future development. To do so, we ask: How do older adults with and without cognitive impairment (including MCI and early-stage dementia) currently use and perceive traditional web search systems and Gen-AI tools, and what factors shape their engagement with these technologies? To our knowledge, this is the first study to directly compare perspectives on traditional web search and Gen-AI systems across healthy older adults, individuals with MCI, and those with early-stage dementia. In doing so, it provides insights to inform inclusive, clinically relevant design of future information systems.

## Methods

### Overview

This mixed methods study explored technology use and opinions of older adults with and without MCI or early-stage dementia. The study was conducted in 2 parts: an online survey and follow-up focus groups. The design accounted for heterogeneity in disease and at-risk populations by including participants across the cognitive spectrum, including early-stage dementia, MCI, and cognitively healthy older adults.

### Ethical Considerations

The study was given ethical approval by the University of Strathclyde Ethics Committee (UEC24/28). All participants provided informed consent prior to participation. Participants were informed of the nature and purpose of the study and their right to withdraw at any time without penalty. All data were collected and stored in accordance with relevant data protection regulations, and no personally identifiable information was included in the analysis or reporting of results. Participants did not receive compensation for their participation.

### Part 1: Online Survey

#### Participants

The survey gathered a total of 325 responses. As some participants did not go beyond the demographic questions, 39 (12%) responses were excluded before analysis, resulting in 286 (88%) valid responses (162 women, 123 men, and 1 who preferred not to say), with a mean age of 72.87 (SD 5.67) years. Partial responses, where participants completed part of the main survey, were included. Participants were recruited via word of mouth, social networking sites (eg, X and LinkedIn), flyers, and the Join Dementia Research platform, with 272 participants identifying as currently residing in England and 14 in Scotland. Education levels are summarized in Table 1. Participants reported diverse professional and vocational backgrounds, including health care, education, government, engineering, business, and skilled trades. This diversity highlights the varied socioeconomic and professional experiences represented in the sample. They were divided into 3 groups: healthy older adults aged older than 65 years without a neurological diagnosis (n=194, 67.8%, mean 72.38, SD 5.55 y; 119 women and 75 men); adults aged older than 65 years with early-stage or mild dementia or MCI (n=87, 30.4%, mean 74.1, SD 5.62 y; 42 women, 44 men, and 1 who preferred not to say); and caregivers of adults aged older than 65 years with early-stage or mild dementia or MCI, who responded on behalf of the person they cared for, providing their demographics (not their own; n=5, 1.7%, mean 70.4, SD 8.47 y; 1 woman and 4 men). Participants with cognitive impairment included 31 (33.7%) with Alzheimer disease, 44 (47.8%) with MCI, 8 (8.7%) with vascular dementia, 3 (3.3%) with frontotemporal dementia, 2 (2.2%) with Lewy body dementia, and 4 (4.3%) with other or unspecified conditions. Due to the small size of the caregiver group, these responses were combined with those of the dementia and MCI group. Sensitivity analyses excluding caregiver responses showed no meaningful changes in results, justifying their inclusion in the combined cognitive impairment group.

**Table 1.** Education level of survey participants^a^.

Education level	Response, n (%)
PhD	16 (5.6)
Bachelor’s degree	73 (25.5)
Master’s degree	31 (10.8)
Some college or further education	88 (30.8)
Secondary school	40 (14)
Other	38 (13.3)

a “Other” includes professional, vocational, or partial higher education qualifications not captured by standard degree categories, such as teaching certificates (Postgraduate Certificate in Education), nursing or medical qualifications, engineering or law certifications, postgraduate diplomas, and other professional credentials.

#### Design

This study was designed as a mixed methods cross-sectional survey using independent samples. The online survey was created and conducted on Qualtrics (Qualtrics International Inc), with questions focusing on respondents’ views of online search systems, such as Google, and Gen-AI systems, particularly conversational Gen-AI, such as ChatGPT.

The survey instrument was developed collaboratively by the first author and 2 supervisors to explore awareness, experience, and perceptions of Gen-AI and online search systems in older adults with and without cognitive impairment (including MCI and early-stage dementia). As no existing validated instruments addressed perceptions of conversational Gen-AI in older adults with cognitive impairment, items were created de novo, and established frameworks such as the Technology Acceptance Model (TAM) were not used because they did not capture the specific nuances of these systems in this population. The survey included a combination of Likert-scale and open-ended questions covering prior use, interaction and question phrasing, perceived response relevance, emotional experience, perceived usefulness, and perceived ability for independent use.

For Gen-AI questions, participants were first asked whether they used this technology. Those who said yes were asked about the applications they use, how often, for how long, and through which devices. They responded to questions on a 5-point Likert scale regarding the ease or difficulty of using the applications and the reasons behind their experiences, their usefulness, ease of selecting and writing queries, the relevance of the output, and their experience with the system in terms of boredom, enjoyment, independence, complexity, engagement, and feeling overwhelmed. Those who said no to using Gen-AI tools were asked about their reasons for not using them, what might encourage them to use these tools in the future, and whether they would consider using them in the future. Most Likert-scale questions were rated between “strongly disagree” and “strongly agree.” These responses were encoded as integers from 1 through 5 and averaged across groups.

Similarly, participants were asked if they used online search systems. Those who answered yes were also asked about which applications they used (specific to general web search engines), how often, for how long, and through which devices. They were given Likert-scale questions about how easy or difficult the applications were to use and why and questions regarding the overall search process. They were asked about query formulation in terms of phrasing and rephrasing questions and relevance assessment regarding whether the search results were relevant, whether enough relevant information was provided, whether they could decide what information was relevant, and whether results were always relevant to their question. They were also questioned about helpful features, any aspects needing improvement, and their experiences with the system, including memory of their search goals, feelings of being lost, engagement, enjoyment, boredom, independence, complexity, and feeling overwhelmed. Those who said no to using online search systems were asked about the reasons for not using them, what might encourage them to use these systems, and whether they would consider using them in the future. All participants were asked at the end of the survey what they would like to see in a system they could design themselves and given an opportunity to add any further comments they wished to voice.

#### Pilot

Five healthy older adults with no cognitive impairments were recruited through the University of Strathclyde Older Adult Research Panel. The pilot sessions were conducted both in person and online. Participants completed the survey with the researcher, providing feedback on the questions, structure, and length of the survey.

Following pilot feedback, minor refinements were made: illustrative images introducing Gen-AI and online search systems were added; it was clarified that “generative AI” referred to conversational, text-based chatbots rather than voice assistants; clarification that questions referred to the search system interface rather than website credibility was included; confusing or redundant response options were removed; and it was more clearly indicated where multiple responses could be selected. Participants reported the survey length and structure to be appropriate, and no major restructuring was required. As the study was exploratory, no formal psychometric validation or reliability testing was conducted.

#### Procedure

Participants first selected the group they identified with, then filled out the relevant participant information sheet and consent form. If they were interested in joining the focus groups, they had the option to provide their email address for future contact. They received an ID and proceeded to the main questions in the survey, completing the *Generative AI* section followed by the *Online Search System* section. A debrief was then displayed. After the survey recruitment, invitations were sent to those who had expressed willingness to participate in the focus groups. Survey completion times were highly skewed due to a small number of very long durations. The median completion time was 825 (IQR 597.5‐1223) seconds.

#### Quantitative Analysis

A series of 2-tailed independent-samples *t* tests were carried out to examine each Likert-scale response between the MCI or dementia and healthy older adult groups. Although Likert-scale data are technically ordinal, independent-samples *t* tests can be considered robust to violations of the interval assumption, particularly for detecting group differences [44]. To account for the small and unequal sample sizes in the Gen-AI comparisons, we conducted supplementary nonparametric analyses using Mann-Whitney *U* tests, which produced results consistent with the *t* tests. Therefore, the reported *t* test results provide a valid representation of group differences.

### Part 2: Follow-Up Focus Groups

Focus groups were organized to explore survey results in greater depth.

#### Participants

Ten survey participants took part in the follow-up focus groups (5 men, 5 women; mean age 73.9, SD 5.67 y). Two (20%) of 10 participants had indicated in the survey that they had used Gen-AI before, and all participants had said yes to having used online search systems. Six (60%) participants identified as healthy older adults, while 4 (40%) had a diagnosis of Alzheimer disease, dysexecutive syndrome due to neurodegeneration, or MCI.

Three focus groups were conducted in total. Group composition was determined based on cognitive status and prior Gen-AI experience: (1) healthy older adults with no prior Gen-AI experience (n=4, 40%), (2) adults with MCI or dementia with no prior Gen-AI experience (n=2, 20%), and (3) a mixed group including both healthy older adults and adults with MCI or dementia, with varying prior Gen-AI experience (n=4, 40%). While group 2 had only 2 participants due to recruitment constraints, their perspectives were included to ensure representation of individuals with cognitive impairment. The characteristics of each group are summarized in Table 2.

**Table 2.** Composition of focus groups.

Focus group	Participants, n (%)	Gender (female/male), n	Age, mean (SD)	Diagnosis^a^	Prior Gen-AI^b^ use^c^
1	4 (40)	3/1	72.5 (6.56)	No impairments	No
2	2 (20)	0/2	71.5 (6.36)	MCI^d^ or dementia	No
3	4 (40)	2/2	76.5 (4.93)	2 MCI or dementia and 2 no impairments	2 yes and 2 no

a Diagnosis refers to the self-reported diagnosis provided by participants in the survey demographic questions.

b Gen-AI: generative artificial intelligence.

c Prior Gen-AI use indicates self-reported experience with Gen-AI tools before completing the survey.

d MCI: mild cognitive impairment.

#### Design

Question topics were informed by initial survey responses, establishing exploration of early patterns, such as trust*,* which was not questioned in the survey, yet was raised in responses. A semistructured interview guide was used to ensure consistent topic coverage across groups while allowing flexibility for probing and follow-up questions. The full interview guide is provided in Multimedia Appendix 1. To minimize moderator bias, neutral questioning was used, participation from all group members was encouraged, and both positive and negative perspectives were explored. The presence of a second researcher allowed oversight of adherence to the interview guide and provided a reflexive discussion following each session. All sessions were audio recorded and transcribed verbatim, and data collection continued until thematic saturation was reached, ensuring rigor and reliability of qualitative findings. The focus groups were moderated by the lead researcher, with a senior supervising researcher present during each session.

#### Procedure

Selected participants were contacted by email for the focus group follow-up. Participants read and completed the participant information sheet and consent form before attending the meeting, and focus groups were conducted online via Zoom (Zoom Communications). Each session lasted approximately 80 minutes, including a short mid-session break. Introductions were made, followed by a short demonstration of Gen-AI, particularly ChatGPT (OpenAI), illustrating the basic functions and capabilities of the system. This demonstration lasted approximately 5 to 10 minutes and followed a scripted sequence of predefined prompts to ensure consistency across groups (Multimedia Appendix 1). The main discussion then centered around Gen-AI, covering overall opinions, experience asking questions, its responses to questions, relevance, trust, and training. The same questions were also posed for online search systems. A brief debrief and a question-and-answer session were provided before closing.

#### Qualitative Analysis

To analyze the qualitative data from both the open-ended survey questions and focus group responses, a reflexive thematic analysis was performed [45]. Themes were iteratively developed and refined across both data sources, allowing the qualitative findings to contextualize and deepen the understanding of the quantitative survey results.

## Results

### Overview

**Figure 1.**
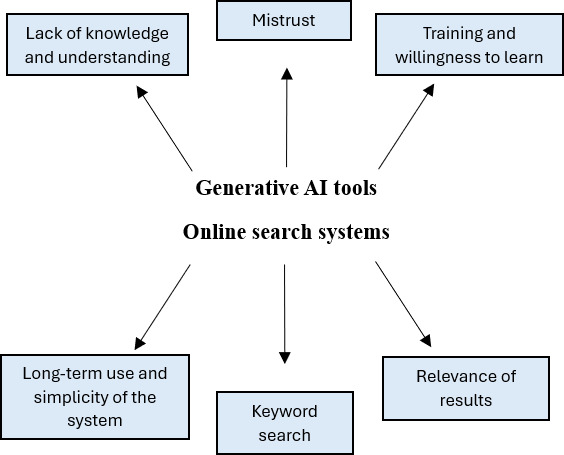
Final themes developed from qualitative data analysis. AI: artificial intelligence.

Thematic analysis of Gen-AI content from the survey open-ended questions and focus groups identified 4 main themes: lack of knowledge about current technologies, mistrust, self-efficacy, and training and willingness to learn. For online search systems, the primary themes were long-term use and simplicity of the system, keyword searching, and relevance of results. Follow-up focus groups explored these themes in more detail, and key findings are presented alongside thematic results (Figure 1).

### Generative AI

#### Quantitative Data

When asked whether participants currently use a Gen-AI tool, 40 (14%) of 286 participants responded “yes,” comprising 27 (13.9%) of 194 older adults and 13 (14.1%) of 92 of those with MCI or dementia, with the most common use being to find information (Table 3). Many participants had heard of this technology, but the reasons given for not having used it varied (Table 4). Despite most participants saying “no” to using Gen-AI, 169 (68.7%) of 246 participants indicated they would consider using Gen-AI tools in the future, highlighting the potential for future adoption. Among those who used these tools, usage was mostly less than monthly, followed by weekly, lasting from a few minutes up to 10 minutes, with ChatGPT being the most frequently used. Thirty-three (94.2%) of 35 Gen-AI users felt that Gen-AI tools could be helpful in their lives. When asked to rate the ease of searching for information using a Gen-AI tool, where 1 was extremely difficult and 5 was extremely easy, the mean rating was 4.15 (SD 0.96).

**Table 3.** Uses of generative artificial intelligence (Gen-AI) and web search^a^.

Uses	Gen-AI, n (%)	Web search, n (%)
To explore	9 (25.7)	139 (53.7)
To find information	22 (62.9)	257 (99.2)
To learn	20 (57.1)	176 (68)
To socialize	1 (2.9)	20 (7.7)
For entertainment	4 (11.4)	81 (31.3)

a Reported use of Gen-AI and web search across participants. N indicates the number of participants who selected each use. Participants could select multiple answers; therefore, totals exceed the number of participants. “Other” and “prefer not to say” were also available options.

**Table 4.** Reasons for nonuse^a^.

Response option	All groups, n (%)	MCI^b^ or dementia, n (%)	Healthy older adults, n (%)
I have heard of it but have never considered using it	113 (45.9)	33 (41.8)	80 (47.9)
I have heard of it but have never had the chance to use it	38 (15.4)	13 (16.5)	25 (15)
I used it and decided to stop using it or didn’t like it	8 (3.3)	1 (1.3)	7 (4.2)
I have never heard of it	87 (35.4)	32 (40.5)	55 (32.9)

a Participant responses for their reasons behind the nonuse of Gen-AI. Percentages are calculated within participants who reported not using Gen-AI (n=246 total; n=79 MCI or dementia; n=167 healthy older adults).

b MCI: mild cognitive impairment.

Independent-samples *t* tests revealed no significant differences between the older adult and MCI or dementia groups, except for independent use of the system, where older adults responded in greater agreement to being able to use Gen-AI independently compared to those with MCI or dementia (Table 5). Nonetheless, there was a noticeable trend of older adults rating items, such as independent use and the ease of use of Gen-AI applications, as more agreeable than the MCI or dementia group. Further investigation through qualitative responses provided additional insights.

**Table 5.** Generative artificial intelligence (AI) Likert responses between groups^a^.

Question	MCI^b^ or dementia, mean (SD)	Healthy older adults, mean (SD)	Significant differences (*P* value)	Effect size (Cohen *d*)
“Generative AI helps me answer my questions”. To what extent do you agree with this statement?	3.77 (1.01)	4.18 (0.73)	.17	−0.49
I have to reword my question to get the answer I’m looking for	3.58 (0.9)	3.36 (1.22)	.59	0.2
I find Generative AI tools simple to use	3.69 (1.11)	4.23 (0.87)	.12	−0.56
I can use Generative AI tools independently	4 (1.15)	4.68 (0.65)	.031	−0.79
I feel overwhelmed when using Generative AI tools	1.85 (1.07)	1.5 (0.86)	.30	0.37

a Responses from participants who reported that they use Gen-AI applications. Full independent-samples *t* test statistics (*t* values, degrees of freedom, and exact *P* values) for all items are provided in Multimedia Appendix 2. Full Likert-scale response distributions are provided in Multimedia Appendix 3. 1=strongly disagree and 5=strongly agree.

b MCI: mild cognitive impairment.

#### Qualitative Data

#### Lack of Knowledge and Understanding

When survey participants were asked why they did not use a Gen-AI tool, responses reflected a general lack of knowledge, such as “I don’t really understand them,” not knowing enough about their potential, such as “don’t really know enough about it, its benefits or how to access and use it,” and not understanding how to operate it, such as “don’t know how to use it.” This also extended to the belief that it is solely for education; one survey participant said:

*...I’ve read it is used for such things as school/University course work... now I’ve seen a full explanation... I will investigate further.*

These qualitative responses align with quantitative trends showing that awareness alone does not guarantee confident adoption. For example, the mean ratings for knowing how to phrase their questions were 3.23 (SD 1.3; MCI or dementia) and 3.86 (SD 1.04; healthy older adults), and for understanding when to use Gen-AI, the mean ratings were 3.67 (SD 1.23) and 4.14 (SD 0.89), respectively (Multimedia Appendix 2). This suggests that limited familiarity may restrict effective use, particularly for the MCI or dementia group. Focus groups provided similar insights, with participant 6 (male, aged 69 y, no impairment) stating, “...I didn’t know what it was and how to use it,” while also expressing interest in using it after hearing about its capabilities.

A minor theme identified in the survey data was the lack of perceived need to use this technology, as reflected by both the MCI or dementia group, “not felt the need,” and in the older adult group, "I have no need to generate tracks of commentary or explanation... I feel confident in my ability to read, assess, and formulate written responses.” Quantitative data support this: although 68.7% of participants indicated they would consider using Gen-AI tools in the future, current use was only 14%. Older adult survey responses offered more detail, with comments such as “have no need to and don’t know enough about it’ and “I’ve not felt a need to use one and I’m not sure how to get started.” These insights suggest that perceived lack of need may reflect limited understanding of Gen-AI applications.

#### Mistrust

A further theme was mistrust. Survey participants who had never used Gen-AI expressed security concerns (“don’t trust it. Think it can be used/hacked for nefarious and cruel purposes”) and concerns about accuracy (“probably foolishly, I don’t feel I can fully trust AI-generated tools or the information they provide”). This aligns with quantitative findings showing lower confidence in independent Gen-AI use (MCI or dementia: mean 4.00, SD 1.15 vs healthy older adults: mean 4.68, SD 0.65; *P*=.03), suggesting that it may limit use. This may relate to limited knowledge about data management.

Survey participants also raised concerns about regulation: “I believe they are dangerous...There is insufficient and inadequate regulation.” This aligns with responses from those who had used Gen-AI but stopped or disliked it; when asked what caused this, survey replies included accuracy concerns, (“suspicious of the accuracy of results”), privacy and security (“I need to be absolutely certain that it’s secure”) and regulation (“better monitoring of these tools by independent third parties”). Quantitative responses showed that although participants generally agreed Gen-AI provides relevant answers (MCI or dementia: mean 3.69, SD 1.32 and healthy older adults: mean 4.09, SD 0.81), lower confidence in phrasing questions (mean 3.23, SD 1.3 vs mean 3.86, SD 1.04) and understanding when to use tools (mean 3.67, SD 1.12, vs mean 4.14, SD 0.89) reflects that mistrust may affect perceived usefulness (Multimedia Appendix 2).

Focus groups discussed trust in more depth. Participants said, “…at the moment I don’t trust artificial intelligence,” and “…I actually trust very little of anything.” Focus group participants also raised privacy concerns:

*I think we have enough observation in our lives… from all the CCTVS… a bit of privacy at your own home is quite nice.*

*I’m concerned as well about these algorithms… it gets to know what you like… that worries me too.*

Regulation was also highlighted: “what is being done with social media and how it’s abused…we allowed that because nothing was regulated,” emphasizing transparency. Participant 3 (female, aged 78 y, no impairment) expressed feeling a lack of control:

*...I don’t know who’s feeding it the information…Where’s that coming from? Is it coming from one source?*

Survey responses mirrored this cautious use: "if I needed to use AI I would, but I prefer not to increase use of AI until I am sure it is controllable.” This mistrust in AI is reflected through sci-fi references by participant 9 (male, aged 74 y, Alzheimer disease), “...these extraordinary machines are not human and all our concerns about sci-fi scenarios in which these machines take over the world,” which again mirrors survey responses, “I think they could be dangerous...watch the Terminator films again. If the machines ever become self aware humanity will be in real trouble.”

Focus groups also highlighted that trusting Gen-AI depends on the user verifying results. Participant 7 (male, aged 82 y, MCI) said:

*if I’ve taken part in history and I quiz the machine... and it responds with a different account... the machine immediately turns around to me and apologizes for providing incorrect facts. So that confuses me a little bit.*

They stressed that their own knowledge is essential:

*if I hadn’t been a witness to the event or taken part in it, I would have believed everything the machine told me...*

Healthy older adults echoed this:

*…results I get back can be hit and miss and you really have to know what it is you’re asking.*

[Participant 8, female, aged 71 y, no impairment]

*AI seems to me to be something that just gives you what it thinks you want rather than what you think you want.*

[Participant 6, male, aged 69 y, no impairment]

Participant 1 (male, aged 76 y, Alzheimer disease) reinforced this:

*if you don’t have the cognitive ability to be able to discern between fact and fiction, then you’re quite likely to be misled, misinformed, or actually harmed.*

These observations help explain why participants reported relevant outputs (MCI or dementia: mean 4.17, SD 0.83), yet lower confidence in independent use, suggesting that mistrust and cognitive concerns influence engagement. Therefore, reliance on the user to identify correct and unbiased information is a significant factor. This was echoed by a participant with MCI or dementia in the survey data, who stated:

*I’m not a fan of AI. I like to be able to tell the difference between what’s real and what’s fake... I also don’t want to be confused as the disease progresses... I would like all AI generated images and text to have to carry an “AI” generated symbol.*

Thus, mistrust and need for oversight appear linked to lower confidence in independent use shown in the quantitative data, highlighting the importance of distinguishing genuine from AI-generated content.

#### Perceived Capability and Self-Efficacy

A distinct theme was participants’ perceived capability when interacting with Gen-AI, reflecting situational self-efficacy. Some focus group participants reported feeling the need to treat AI as more capable than themselves. For example, participant 7 (male, aged 82 y, MCI) said:

*...I couldn’t treat Gemini or GPI as a machine... For me to relate to them and to use them, I had to treat them as human beings who were more intelligent and more verbal than I was.*

Conversely, focus group participant 8 (female, aged 71 y, no impairment), who uses Gen-AI, offered a contrasting viewpoint, “...I believe I treat it as a machine, as a supercomputer.” These perspectives contextualize survey responses reporting discomfort with interactions, “the robotic nature of the exchanges and limitations to interact are uncomfortable to deal with,” and may help explain variance in engagement and trust metrics across participants. Although perceived capability was not directly measured, these qualitative insights suggest that participants’ situational self-efficacy in using Gen-AI varies depending on how they perceive the AI’s abilities relative to their own.

#### Training and Willingness to Learn

When asked about what would encourage participants to use Gen-AI in the future, survey responses reflected training needs and a general willingness to learn. The MCI or dementia survey group often requested “more knowledge, experience, and training” and “an explanation and teaching of how to do this.” Similar responses were given by the older adult survey group, expressing a desire for information on “how it would benefit me followed by training on how to use it,” including “a demonstration and practice in using them” and access to “free training sessions possibly at a library.” Both survey groups also mentioned the importance of support from others to encourage use, such as “having someone around with the time to explain it and then help me to use it” in the MCI or dementia group, and “recommendation by a friend or family member...” in the healthy older adult group. Quantitative data support this: 68.7% (169/246) of survey respondents indicated they would consider using Gen-AI in the future. Additionally, Likert-scale responses showed moderate agreement that Gen-AI helps answer questions (MCI or dementia: mean 3.77, SD 1.01; and healthy older adults: mean 4.18, SD 0.73) and provides relevant answers (MCI or dementia: mean 4.17, SD 0.83; and healthy older adults: mean 4.18, SD 0.73). This suggests that addressing knowledge gaps and providing transparency could facilitate adoption.

Survey participants also outlined their expectations for training, including desires for “a clear idea of what it can do and be used for,” “more information and guidance in how to use and its benefits,” and practical examples. Comments also reflected concerns about trust and system performance. The MCI or dementia group mentioned, “only if I could be absolutely assured that what was produced was true and could be collated with other trusted sources of information,” while the older adult group expressed concerns about accuracy, stating, “being reassured that they don’t make up the answer if they can’t find it.” These concerns parallel survey Likert items on confidence in independent use, where lower ratings among participants with MCI or dementia (mean 4, SD 1.15 vs 4.68, SD 0.65 in healthy older adults) may reflect caution about trusting outputs and the need for training to build confidence while addressing reliability and accuracy.

Despite encouraging responses, it is important to acknowledge that some reactions showed reluctance to engage. Some participants replied “nothing” when asked what would encourage them to use these systems, while others, such as from the MCI or dementia group, expressed, “I personally don’t feel I need anything like this, at the moment,” and “Nothing. I do not trust the security of these sites.” Older adults also shared perceptions that there is too much technology, with comments such as “I don’t want to get involved with anything else,” and believed that web search was sufficient, stating “I don’t think I need to use such a tool, as I can find most information I need on Google.” Willingness to use Gen-AI appears shaped by personal beliefs and experiences, with one noting, “I believe this tool is used to help create content/write something...I have never felt the need to use it.” Some responses from the MCI or dementia group cited complexity, “it’s too complicated,” “I’m not very good with tech stuff,” “coping with technology,” and “I am a technophobe.” These hesitations correspond with quantitative patterns, such as lower confidence in phrasing questions (MCI or dementia: mean 3.23, SD 1.3 vs healthy older adults: mean 3.86, SD 1.04) and understanding when to use Gen-AI (3.67, SD 1.23 vs 4.14, SD 0.89). This indicates that not all barriers can be addressed through training alone.

Both groups also noted ease of use and user-friendliness: “usefulness outweighing frustration.” When asked about ease of use, those who used Gen-AI responded positively. The MCI or dementia group said, “...it provides a response that I understand,” “it is very responsive to questions posed to it. I often put in further questions...,” and “I am just starting with AI as I feel it could be of great help as my condition progresses.” Older adults responded similarly, “the info is quite detailed but is easy to query in more detail by using more targeted questions,” “no harder than searching in Google,” and “a bit better and precise than an internet search.” However, some negative points were raised, such as, “with some questions the AI has to be tricked into answering, or will not answer,” and concerns about complexity: “I have limited tech” experience and, as I have no family or support network, I can be slow to learn & retain new methods.” Overall, these experiences align with Likert-scale ease of use scores (mean 4.15 overall).

The focus group participants expressed varied interest in training. Participant 2 (male, aged 67 y, dysexecutive syndrome) said, “I would go and get that training because I’m curious...the problem is being able to remember... what you’ve learned,” highlighting that training might be most effective early in diagnosis or beforehand. Participant 1 (male, aged 76 y, Alzheimer disease) expressed a preference for being taught by similar individuals, stating:

*...training would be most effective if- if it was done by people such as myself who were in a MCI state and would have an understanding of what it was that they were talking about…*

Others, who were more skeptical about AI, did not show interest in training, “I just think it’s money wasted on me at 80... better given the training to the youngsters so they’re prepared for the future rather than me,” or simply conveying a lack of interest in attending. Participant 10 (female, aged 79 y, no impairment) expressed a preference to explore it in their own time, stating:

*I don’t think in my case I would need training. I think I would look at it and- and explore it on my own.*

Additionally, it was discussed that targeted training for groups could be most beneficial:

*I think that training on Gen-AI as a whole for members of the general public would probably possibly not be that useful...*

Despite varied interest, some participants found the focus group demonstration intriguing, saying:

*when you were givin’ the demonstration of ChatGPT, I wanted to start using it myself to, to find some information that I’ve not been able to get easily from Google.*

Overall, responses highlight that individual experiences, cognitive abilities, and confidence shape willingness to learn, but there is potential for training to help those interested.

### Online Search Systems

#### Quantitative Data

Participants were asked if they currently use an online search system. Of the 286 survey participants, 280 (97.9%) responded to this question, reflecting partial survey completion. Among respondents, 275 (98.2%) of 280 participants responded “yes,” with 186 (98.9%) of 188 older adults and 89 (96.7%) of 92 participants in the MCI or dementia group reporting use. Among these users, Google was the most used, and most participants reported using search systems daily and for less than 5 minutes at a time. Participants used these tools primarily to find information and learn (Table 3). When asked how easy they found searching for information using an online search system, where 1 was extremely difficult and 5 was extremely easy, the average response was 4.38 (SD 0.8), indicating generally positive perceptions.

Independent-samples *t* test results reveal significant differences between groups on several survey questions, with the healthy older adult group consistently rating items as more agreeable (Table 6) than the MCI or dementia group. For example, the MCI or dementia group was more likely to report needing to reword queries, feeling lost, or feeling overwhelmed, whereas healthy older adults more often reported being able to remember what they were searching for, deciding whether information was relevant, and using search systems independently. Survey open-ended questions and focus groups provided additional insight.

**Table 6.** Web search Likert responses between groups^a^.

Question	MCI^b^ or dementia, mean (SD)	Healthy older adults, mean (SD)	Significant differences (*P* value)	Effect size (Cohen *d*)
I have to reword my question to find what I am looking for	3.35 (1.1)	2.94 (1.09)	.005	0.37
I can decide if information is relevant to my question	4.3 (0.74)	4.57 (0.54)	.005	−0.42
I can remember what I was searching for during the search process	4 (1.09)	4.8 (0.44)	<.001	−1.14
I feel lost during the search process	2.39 (1.2)	1.53 (0.85)	<.001	0.88
I can use online search systems independently	4.32 (0.97)	4.67 (0.71)	.003	−0.44
I feel overwhelmed when deciding which information is relevant	2.54 (1.38)	1.7 (1)	<.001	0.74

a Responses from participants who reported that they use web search. Full independent-samples *t* test statistics (*t* values, degrees of freedom, and exact *P* values) for all items are provided in Multimedia Appendix 4. Full Likert-scale response distributions are provided in Multimedia Appendix 5. 1=strongly disagree and 5=strongly agree.

b MCI: mild cognitive impairment.

#### Qualitative Data

##### Long-Term Use and Simplicity of the System

Survey participants were asked about why using these systems was easy or difficult. A prominent response was prior experience, reported by both the MCI or dementia group (“I have a background in IT...”) and the healthy older adult group (“I have used computers in my last job for many years”). Similarly, both survey groups reported personal use, “I have used it for many years, so have been familiar with it for a long time.” These findings align with the quantitative results showing high ease of use (mean 4.38, SD 0.8 overall) and reported high levels of independent use, particularly among healthy older adults (mean 4.67, SD 0.71 vs MCI or dementia mean 4.32, SD 0.97; *P*=.003), suggesting that familiarity underpins perceived ease. Moreover, survey responses often mentioned web search being habitual, “having used various search engines over many years it is almost second nature,” also consistent with quantitative findings of daily use and low feelings of overwhelm when inputting queries (MCI or dementia: mean 2.08, SD 1.24 vs healthy older adults mean 1.34, SD 0.79; *P*<.001). Focus group participants echoed this, noting challenges for learning AI: “I haven’t used it (referring to Gen-AI) because I’m a creature of habit... if I want an answer to something I Google it,” and “one of the necessary keys to people with memory problems in using new technology like AI is that we are people of habit. And that’s going to be the big, the big stumbling block.”

Some survey responses indicated that the enjoyment of discovering information facilitated ease of use, “I enjoy finding things out and have learned how to do this.” Notably, one MCI or dementia survey participant said, “the information is there to be found now that I have learned how to access it. Having family to help me would have been game changing but I have managed. I am acutely aware that as any tech’ ability I have learned… I shall lose these first, so lose this invaluable source of managing my life as Alzheimer’s progresses.” This highlights the impact of IR on independence and daily living and corresponds with survey findings showing lower feelings of being lost among healthy older adults (mean 1.53, SD 0.85) compared with the MCI or dementia group (mean 2.39, SD 1.2; *P*<.001), emphasizing the role of experience in maintaining confidence during searches.

##### Keyword Searching

Keyword searching was discussed in response to questions about ease of use, with survey participants describing it as both easy and difficult. Some participants with MCI or dementia found it straightforward: “I have always been good at asking questions,” while others found it more challenging: “it depends what I’m looking for. Finding the right words isn’t always easy. The more branches I go down, the more difficult I find it to get back,” and “the most frustrating aspect is when I am unable to select a search term that brings up correct answers... I’m only offered a limited selection of answers and I know there must be more but they don’t appear..." Consequently, some mentioned, “I sometimes have to change my question,” reflecting the quantitative finding that the MCI or dementia group responded significantly more strongly with needing to reword questions (mean 3.35, SD 1.1) compared to the healthy older adult group (mean 2.94, SD 1.09; *P*=.005; Table 6).

Healthy older adults generally reported less difficulty: “simple to phrase a question,” and that rephrasing, if needed, was straightforward: “can usually find what I want quickly but do need to consider how a question is phrased,” aligning with quantitative findings that healthy older adults had greater confidence in phrasing questions (mean 4.2, SD 0.73 vs MCI or dementia mean 3.9, SD 1.03; *P*=.02). Both groups highlighted how keywords influence search results, with comments such as “depend on selecting suitable key search words, and the main problem is evaluating the reliability of sources of information...” and “with Google there are a lot of irrelevant web pages returned and it’s often necessary to search through the links to find what you are looking for.” Overall, there is a contrast between finding keyword searches easy or difficult across both groups, with an acknowledgment that the relevance of results often depends on this.

Focus group participants provided context for these survey patterns. Many participants reported that searching was easy, but occasionally required rephrasing. Participant 5 (female, aged 65 y, no impairment) said:

*“I think I’ve always found what I wanted,” adding, “...if the results are not relevant... I know I need to rephrase the question...”*

Participant 6 (male, aged 69 y, no impairment) mentioned that their frustration with web search makes Gen-AI appealing:

*I wanted to start using it myself to- to find some information that I’ve not been able to get easily from Google...I think it could focus when I want a question answered...*

Overall, survey and focus group data indicate variability in ease of keyword searching, with rephrasing often necessary and keyword selection affecting relevance. Participants with MCI or dementia experienced more difficulty, reflecting quantitative differences.

##### Relevance of Results

Another identified theme was the relevance of results, particularly concerning advertisements, sponsorships, and location bias. Survey participants highlighted that commercialization reduces relevance, with the MCI or dementia group noting, “it would be easier if the results of searches didn’t have such a converging and commercial aspect,” and the older adult group stating, “the answers it gives are too random and the paid answers are always crowding out the simple ones.” Both groups also reported irrelevant information: “too much irrelevant information given in answers,” and “...I am not always convinced by the accuracy or bias in the info I find...” Suggested improvements from survey responses included: “by not showing promoted content first which is irrelevant to my question,” “ditch the cookies, by the time I’ve got through this it’s hard to remember what I was thinking about,” and “less emphasis on American English terminology, and less bias towards American based info/research.” Survey comments also suggested implementing “color-coded hyperlinks” to better distinguish between promoted and relevant results and that “search engines should organize their results so as to assist the searcher rather than according to commercial and financial priorities.”

Expanding on the survey concerns, dislike for commercialization was also observed in the focus groups, where participant 5 (female, aged 65 y, no impairment) said, “...the first one that always will come up in the list is sponsored,” while participant 3 (female, aged 78 y, no impairment) added, “it disturbs me that...the people who pay most money get up the queue...” This was also linked to trust, with participant 1 (male, aged 76 y, Alzheimer disease) noting that commercial prioritization can facilitate harm:

*...they will draw revenue from people who are willing to pay to have their entry first in the list... there are a lot of people who get misled.*

Participants also described strategies to reduce risk, such as using privacy-focused browsers, “I use DuckDuckGo because it removes trackers …it just makes me feel more comfortable,” although survey responses indicated a knowledge gap: “I wish there was a way of feeling safer when I do the searches.”

When survey participants were asked what they would include if they could design their own system, responses addressed relevance filtering, source transparency, and removal of sponsored results, “...A button to click that would identify owners/contributors..,” “1. Removal of sponsored responses; 2. Dating of posts to allow disregarding of outdated posts; 3. It may become necessary to identify AI responses derived from old or fake data that should not be relied upon.” Interestingly, Gen-AI was suggested as a potential improvement to current systems by the older adult survey group, with comments such as "ChatGPT is useful because you can build a series of questions on a topic to look at different aspects of an issue and then draw on the answers to build a summary,” and the MCI or dementia survey group, who said, “I manage my online searches quite easily as long as I am careful with my phrasing. AI is able to help me with that.” Others acknowledged that these systems could be improved, stating, “I only know they could be better for me.”

Finally, focus group data suggested that the volume of results can affect focus, particularly for participants with MCI or dementia. Participant 7 (male, aged 82 y, MCI) said, “if I ask Google a specific question... I would like a specific answer,” whereas participant 3 (female, aged 78 y, no impairment) appreciated the element of choice, saying, “Google gives me choices so I can go in and I consult there.” This may be reflected in the survey responses from the MCI or dementia group, which mentioned, “keeping what I am searching for in mind, sometimes I forget mid-search,” and “remember what I am doing, short span of concentration, tired easily.” The higher volume of results may impose a greater cognitive load or memory pressure when users must determine the most relevant result to select. This may be consistent with quantitative results depicting the MCI or dementia group rating being able to remember what they were searching for as lower (mean 4, SD 1.09) compared to the healthy older adult group (mean 4.8, SD 0.44; *P*<.001)*.*

### Participant Perspectives on Future Applications of AI

Building on the survey finding that 68.7% (169/246) of respondents reported openness to using Gen-AI in the future, this theme was explored primarily through qualitative data to understand how participants envisioned AI supporting future daily living needs. Specifically, many envisioned AI systems evolving beyond IR toward proactive monitoring, routine support, and safety. Survey responses acknowledged this:

*happy with search but still trying to get to grips with AI which I feel has tremendous potential for people with my complaint.*

The MCI or dementia survey group mentioned a monitoring feature:

*...AI would be ideal to monitor my behaviors...I would be quite happy for my AI to learn all about me and monitor the changes in my behaviors and routine.*

This was taken further in the same response, discussing how their conversational ability could be helpful to them: “...a bit of conversation is helpful when you live on your own!”

These future-oriented applications were not captured in the survey Likert-scale items and were therefore explored in more depth within the focus groups. Participant 4 (female, aged 78 y, no impairment) said, “...I live alone and at the moment I’m hale and hearty but I could have a camera and my daughter could see what I was up to...Things like that I think are good,” indicating that monitoring might be accepted as part of a care plan. This was also discussed extensively by participant 1 (male, aged 76 y, Alzheimer disease) who explained:

*I’m quite happy to share my personal antisocial habits with a computer if it is looking after me...*

This suggests that the potentially adaptable monitoring abilities of AI could be accepted if it improved independent living. However, they also explained that this technology would need to be implemented early in their diagnosis so it could develop alongside them: “...the technology and the help can grow with the disease as it progresses down the step ladder,” the step ladder referring to diminishing cognitive decline, “you’ve got to grow into it... it’s got to be led by the individual’s needs.” Interestingly, this conflicts with concerns about trust and privacy; when asked directly, their perspective on trust was different:

*by the time my disease develops to the point where I’m going to need AI to look after me, my mind will have degenerated to such a state that I actually don’t give two hoots who knows or cares or anything else...I won’t have any privacy anyway, so that is really not of a major concern to me.*

A similar stance was taken by participant 5 (female, aged 65 y, no impairment), reflecting on a different meeting about robots used in care:

*we all went into this meeting saying no way on earth would we have a robot for this. And we all left the meeting saying yes, absolutely, if it meant that we could stay at home...*

Finally, a few notable features were discussed in the focus groups. The first was that AI should learn to forget information, “necessity for AI to learn to forget... when it comes to bank details, jobs in assembly, there are things I think that would be good for AI to recognise it needs to forget…,” and proactive autonomy, “AI responds to requests but what AI doesn’t do is, is have the autonomous ability to, to, to access prompts ...if AI could be trained to think autonomously about where the needs are and what prompts are—that would be a major step forward in helping people with dementias and brain damage to live independently.”

## Discussion

### Principal Findings

To our knowledge, this is the first study to gather in-depth perspectives of older adults with and without MCI (including MCI and early-stage dementia) on their engagement with and opinions of both traditional IR systems and Gen-AI. This contributes new empirical evidence to the literature on digital engagement in older adults, as most prior studies focus either on younger populations or on health information seeking alone [11,46]. Results show that most participants reported using traditional web search systems, consistent with the understanding that digital literacy in this population is growing [47,48]. Participants were recruited via convenience sampling, which may overrepresent older adults with higher digital literacy or motivation to engage with technology; findings should therefore be interpreted as indicative rather than representative of the broader population. Our findings confirm prior work suggesting that habitual use and familiarity enhance perceived ease of use among older adults [49,50], while also extending this by showing differential experiences in individuals with cognitive impairment. This familiarity likely reflects the long-established nature of web search platforms [12], whereas Gen-AI tools are newer and less commonly adopted, consistent with observations that novelty and lack of exposure limit adoption in older populations [51]. As a result, participants reported greater confidence and skill when using web search compared to Gen-AI. Together, these findings address our aim of examining how these groups use and perceive web search and Gen-AI tools.

Both groups generally found web search helpful, easy to use, and understood when and how to use it. Likert-scale responses showed significant group differences across multiple aspects of the search process, with effect size estimates ranging from moderate to large, indicating meaningful differences in user experience. The MCI or dementia group was more likely to report needing to rephrase questions, feeling lost, and feeling overwhelmed, whereas healthy older adults more often reported knowing how to phrase questions, finding relevant information, remembering search goals, and using systems independently. These findings confirm earlier literature showing that cognitive impairment can increase effort and reduce confidence in information-seeking tasks [52,53] and extend it by quantifying the differences in self-reported behaviors and perceptions. Although overall overwhelm remained low, results suggest that cognitive impairment may increase effort during information seeking [52]. Despite group differences, quantitative data depicted that web search was generally viewed positively [54], while qualitative data provided deeper insight into these high ratings, highlighting issues such as commercialization, privacy concerns, and keyword selection, which can impact the user experience.

This adds qualitative insight to existing quantitative findings on older adults’ web search behavior [52]. Qualitative responses reflect individual struggles based on beliefs and abilities, indicating that cognitive impairment shapes the subjective experience of IR systems [53,55,56]. Participants’ proposed solutions indicated that challenges with web search are widespread and may be mitigated through Gen-AI features, with suggestions such as Gen-AI offering more personalized and adaptive responses. These findings complement research on adaptive AI for older adults [57,58] and highlight potential applications supporting independent information access. These findings extend the literature primarily focused on online health information quality [59-61] by highlighting the experiential differences and potential technological improvements for older adults with and without cognitive impairment (including MCI and early-stage dementia). They further suggest that emerging technologies such as Gen-AI may help offset age–related and cognition-related disparities in access [42,62].

Regarding Gen-AI, although reported usage was lower, more than half of nonusers expressed willingness to use these tools in the future, indicating potential uptake if barriers are addressed. Adoption appeared to be influenced by beliefs [51], including mistrust and lack of knowledge, consistent with prior studies showing that trust and perceived knowledge influence technology adoption among older adults [39,63-67]. However, qualitative findings suggest that low engagement cannot be attributed solely to knowledge gaps or mistrust. Some participants reported “no need” for these tools, reflecting perceptions of limited personal utility or preference for existing methods, while others identified practical barriers, such as complexity, navigation difficulties, access limitations, and reliability concerns. These observations extend adoption models [68], suggesting that perceived usefulness and interface design may be equally important in older adult populations.

These findings can be interpreted through the lens of the TAM [69], which proposes perceived usefulness and ease of use as key determinants of adoption. Participants recognized potential usefulness but reported limited adoption due to unfamiliarity and perceived complexity, indicating that both constructs were influential. Applying TAM situates these qualitative insights within an established theoretical framework [19,69]. Together, results suggest that utility, accessibility, design, trust, and perceived relevance interact to shape engagement with Gen-AI among older adults and those with MCI or early-stage dementia. This aligns with established links between technology adoption and trust [39,63-67,70]; however, actual uptake was not measured, and further work is needed to determine whether intentions translate into sustained engagement.

Our findings also highlight an important nuance related to participants’ perceived self-efficacy when engaging with Gen-AI. Some focus group participants described feeling less capable than the AI, while others framed their interaction as treating it as a machine. These results extend literature on self-efficacy theory and technology adoption [71-73] by showing that perceived capability may influence engagement, a factor not typically emphasized within TAM. Even when participants recognized usefulness, confidence appeared shaped by perceived competence relative to the AI, particularly among individuals with cognitive impairments, who may already have lower baseline self-efficacy in technology use [72-74]. These perceptions suggest that Gen-AI may unintentionally exacerbate feelings of reduced capability for some users, despite recognized benefits. This highlights the importance of supportive interfaces, gradual exposure, and tailored training to strengthen self-efficacy. Our findings reinforce prior research [19,43] highlighting both barriers and quality of life benefits associated with AI adoption among older adults, while illustrating mechanisms through which Gen-AI may support independent living. Participants’ willingness and motivation to learn indicate promise, although prior work shows that intention does not always translate to adoption [51,74,75]; therefore, findings should be interpreted as reflecting potential engagement rather than confirmed behavior change.

Knowledge gaps were common across both groups, indicating that education and training may support adoption. Participants expressed interest in tuition and demonstrations, particularly for ChatGPT [76], aligning with training practices already recognized [30]. However, views were heterogeneous: some participants welcomed structured training, while others viewed it as unnecessary or of limited value. These findings suggest that one-size-fits-all approaches may be ineffective and that training should be flexible and responsive to individual needs and motivation [77,78]. Tailored or optional learning approaches may therefore better support diverse preferences and abilities. Successful interventions may need to balance guidance with autonomy, allowing access to training without assuming universal interest. Structured, transparent education may improve confidence and understanding while complementing web searches and addressing perceived limitations.

Notably, the MCI or dementia group highlighted issues not raised by healthy older adults, including forgetting mid-search, difficulties learning new skills, and challenges in adopting new technologies. They also rated items such as being able to use Gen-AI independently with lower agreement compared to their older adult counterparts. These findings suggest that early introduction of AI and the inclusion of diverse cognitive groups in design processes are crucial. The adaptability and personalization of Gen-AI may help address these challenges [20], potentially supporting independent living outcomes.

Although only one group difference was statistically significant, effect size estimates indicated moderate-to-large differences across measures related to confidence, usability, and independence. This suggests that participants with MCI or dementia may experience greater difficulty when engaging with Gen-AI tools. However, moderate-to-large effect sizes alongside nonsignificant findings likely reflect limited statistical power due to the small number of Gen-AI users, and the observed effect sizes may help inform future power analyses and sample size targets in subsequent studies.

Furthermore, an emerging factor was monitoring, with participants recognizing AI’s potential to support daily functioning. This aligns with research showing that chatbots may assist in monitoring and informing care providers about symptom changes [79]. Our findings extend this literature by demonstrating that older adults themselves view monitoring as valuable when independence benefits are clear. Despite privacy concerns, focus group responses expressed willingness to adopt AI if the benefits to independence were substantial. These findings highlight the importance of involving older adults in technology development, especially to address perceptions that AI is unnecessary or irrelevant to them. This is reflected in the theme of “no need,” which appears linked to limited awareness of potential benefits [41], yet participants offered clear suggestions for how AI could improve daily life. Therefore, future developments should prioritize early implementation and co-development with older adults and those with cognitive impairment to ensure relevance and utility. Finally, these results complement literature showing that Gen-AI may reduce loneliness, support mental health, and improve person-centered information access, while reinforcing barriers such as ethical concerns, ageism, and digital literacy gaps [17,80]. Our findings demonstrate how these perceptions vary by cognitive status and identify actionable areas for intervention and training.

### Limitations

The dementia and MCI groups were smaller than the healthy older adult group, which may limit representativeness. Additionally, one focus group included only 2 participants due to recruitment constraints; although the discussion generated rich data, larger groups may provide broader interactional insights in future research. Furthermore, participants were recruited through convenience sampling, which may overrepresent individuals with higher digital literacy or technology interest. Similarly, the number of current LLM users was small, although this reflects real-world usage patterns. Surveys were conducted online, restricting control over responses and preventing follow-up clarification, although focus groups helped mitigate this.

While demographic information such as age, gender, education level, occupation, and geographic location was collected, ethnicity and detailed socioeconomic indicators were not measured. This limits generalizability, as cultural and socioeconomic factors may influence engagement with web search and Gen-AI tools. Future research should recruit more diverse samples to explore these influences among older adults with and without cognitive impairment (including MCI and early-stage dementia).

### Conclusions

Our results provide novel insights into how older adults with and without MCI or early-stage dementia may perceive and engage with web search and Gen-AI tools, addressing an underexplored gap in user experience research [81]. Participants expressed interest in Gen-AI, highlighting areas where training and support may facilitate adoption. While findings reflect perceptions and intentions rather than confirmed behavioral change, they identify factors influencing engagement with Gen-AI. They offer a flexible, personalized approach to information access and conversational search, which may help address limitations identified in web search systems. While web search remains valuable due to its long-standing use, participants highlighted areas for improvement, suggesting that hybrid or complementary approaches with Gen-AI could improve accessibility. The study contributes to an underexplored area of literature where potential applications have been recognized [20,82-84] and aims to inform future technological development grounded in user perspectives.

### Future Work

As Gen-AI adoption grows, future research should explore targeted training interventions following best practices [30] to enhance adoption, confidence, and digital literacy among older adults with and without cognitive impairment (including MCI and early-stage dementia). Longitudinal research combining usage tracking and qualitative interviews may clarify evolving perspectives and sustained engagement. Expanding this research to include other underrepresented populations and conditions would be valuable for broadening understanding and assessing the generalizability of the findings. Developers should actively co-design technologies with these groups to combat digital ageism, ensure accessibility, and promote person-centered AI systems. Moreover, effectively evaluating how Gen-AI is integrated into these technologies will be essential to maximize their impact.

## Supplementary material

10.2196/85626 Multimedia Appendix 1 Focus group interview guide and ChatGPT demonstration script.

10.2196/85626 Multimedia Appendix 2 Responses from participants who reported that they use generative artificial intelligence applications (1=strongly disagree and 5=strongly agree).

10.2196/85626 Multimedia Appendix 3 Generative artificial intelligence Likert-response distributions.

10.2196/85626 Multimedia Appendix 4 Responses from participants who reported that they use web search ( 1=strongly disagree and 5=strongly agree).

10.2196/85626 Multimedia Appendix 5 Web search Likert-response distributions (%).

## Abbreviations

AI: artificial intelligence
Gen-AI: generative artificial intelligence
IR: information retrieval
LLM: large language model
MCI: mild cognitive impairment
TAM: Technology Acceptance Model
