# Supplementary material for: Exploring Information Access in Aging Populations and Those With Dementia and Mild Cognitive Impairment in the United Kingdom: Survey and Focus Group Study
Source: JMIR Aging. 2026 Apr 21;9:e85626. doi: 10.2196/85626 (PMC13099020; doi:10.2196/85626)
Supplement: Multimedia Appendix 1 [file aging-v9-e85626-s001.docx]

# Focus group interview guide and ChatGPT demonstration script.

At the start of each session, participants were reminded that participation was voluntary, that there were no right or wrong answers, and that confidentiality of group discussions should be respected. Sessions were conducted online via Zoom and audio-recorded for transcription.

Each session began with a brief moderator introduction, participant introductions, and an overview of housekeeping rules. The session then followed the structure below:

1. Standardised demonstration of ChatGPT
2. Semi-structured discussion of Generative AI
3. Short break
4. Semi-structured discussion of Online Search
5. Closing questions and debrief

| **Standardised ChatGPT demonstration script** (5–10 minutes) |
| --- |
| Participants were asked if they use Generative AI tools, and if not, had they heard of Generative AI tools like ChatGPT before the demonstration. The following scripted prompts were used in every focus group to ensure consistency across sessions. The moderator shared their screen and entered each prompt live into ChatGPT. |
| **Demonstration prompts:**  1. “Hi, how are you?”  2. “How is your day going?”  3. “What are the steps to using an air fryer oven?”  4. “What is the internet?”  5. “Please write it in simpler terms.”  6. Follow-up clarification based on ChatGPT’s response, for example:   - “What is a server?” - “What is a protocol?”   7. “Please write this in bullet points.” |
| **Purpose of demonstration**  This demonstration was designed to show:   - Conversational interaction - Asking general questions - Requesting step-by-step instructions - Simplifying responses - Asking follow-up questions - Changing response format |
| Participants were then invited to suggest additional questions if desired. |

## Semi-structured interview guide

### Section 1: generative ai (ChatGPT)

**Awareness, experience, and usefulness**

Have you heard of generative AI tools such as ChatGPT or Gemini?

- Have you used any of these tools before?

- If yes: What have you used them for?

- What did you like or dislike?

- Were they easy to use?

- What could be improved?

- If no: Would anyone be open to talking about why they haven’t used one before?

- What are your overall opinions on this kind of technology? What do you think?

- Is there anything specifically stopping you from using them?

- What do you think these could be used for in your life?

- Is there something that would make it better or more useful for your daily activities?

- What would it need to be able to do to encourage you to use it?

**Question phrasing and interaction**

Gen-AI doesn’t require keywords like Google does. Does anyone have any thoughts on whether the conversational or natural way you ask a question is positive or negative?

- How do you find phrasing questions to a generative AI tool?
- Have you ever had to reword a question? Tell me more.
- Are these tools simple or complex to use? Why?

**Responses and relevance**

What do you think about the answers these tools provide?

- Do you feel the information is relevant to your questions?

**Emotional experience**

How would you describe your experience using these tools?

- Do you feel overwhelmed at any point?
- Do you find them engaging or interesting?

**Trust**

What are your thoughts on trusting generative AI tools?

**Training and support**

Has training ever been offered to you for generative AI tools?

- If yes: explore

- If no: Would training encourage you to use them?

- What should training include specifically?

- Would training be of interest to you if it were made available?

- What would discourage you from attending?

- What would make training accessible and useful?

**Independent use**

Do you think you could use these tools independently?

What would need to be in place to support independent use?

**Closing** - Is there anything else you would like to add about generative AI tools?

[break]

### Section 2: online search systems (e.g., Google)

**Awareness, experience, and usefulness**

Do you use online search systems such as Google?

- If yes: What do you use them for?
- What did you like or dislike?
- Were they easy to use?
- What could be improved?
- If no: Would anyone be open to talking about why they haven’t used one before?
- What are your overall opinions on this kind of technology? What do you think?
- Is there anything specifically stopping you from using them?
- What do you think these could be used for in your life?
- Is there something that would make it better or more useful for your daily activities?
- What would it need to be able to do to encourage you to use it?

**Question phrasing and keywords**

How do you find phrasing questions in online search systems?

- Have you had to reword questions?
- What are your thoughts on keyword-based searching? Tell me more.
- Are these tools simple or complex to use? Why?

**Responses and relevance**

What do you think about the answers these tools provide?

- Is the information returned relevant to your question? Is it always relevant?
- Do you ever feel lost when using search systems?

**Emotional experience**

How would you describe your experience using online search systems?

- Do you feel overwhelmed at any point?
- Do you find them engaging or interesting?

**Trust**

What are your thoughts on trusting online search systems?

**Training and support**

Has training ever been offered to you for online search systems?

If yes: explore

- If no: Would training encourage you to use them?

- What should training include specifically?

- Would training be of interest to you if it were made available?

- What would discourage you from attending?

- What would make training accessible and useful?

**Closing** - Is there anything else you would like to add about online search systems?

## End of session

Participants were thanked for their time. A brief debrief and question-and-answer session was provided before closing each focus group.
