# Supplementary material for: Exploring Information Access in Aging Populations and Those With Dementia and Mild Cognitive Impairment in the United Kingdom: Survey and Focus Group Study
Source: JMIR Aging. 2026 Apr 21;9:e85626. doi: 10.2196/85626 (PMC13099020; doi:10.2196/85626)
Supplement: Multimedia Appendix 2 [file aging-v9-e85626-s002.docx]

Generative AI Likert responses between groups^a^.

| Question | Group | n | Mean±SD | t | Df | *P* | Cohen’s d |
| --- | --- | --- | --- | --- | --- | --- | --- |
|  |  |  |  |  |  |  |  |
| **“Generative AI helps me answer my questions”. To what extent do you agree with this statement?** |  |  |  |  |  |  |  |
|  | MCI/Dementia | 13 | 3.77±1.01 | -1.4 | 33 | .17 | -.49 |
|  | Healthy Older Adults | 22 | 4.18±.73 |  |  |  |  |
| **The answer Generative AI tools give me is relevant to my question** |  |  |  |  |  |  |  |
|  | MCI/Dementia | 12 | 4.17±.83 | -.06 | 32 | .96 | -.02 |
|  | Healthy Older Adults | 22 | 4.18±.73 |  |  |  |  |
| **I have to reword my question to get the answer I’m looking for** |  |  |  |  |  |  |  |
|  | MCI/Dementia | 12 | 3.58±.9 | .55 | 32 | .59 | .2 |
|  | Healthy Older Adults | 22 | 3.36±1.22 |  |  |  |  |
| **I know how to phrase my question to a Generative AI tool** |  |  |  |  |  |  |  |
|  | MCI/Dementia | 13 | 3.23±1.3 | -1.59 | 33 | .12 | -.56 |
|  | Healthy Older Adults | 22 | 3.86±1.04 |  |  |  |  |
| **Generative AI tools provide relevant answers to the question I am asking** |  |  |  |  |  |  |  |
|  | MCI/Dementia | 13 | 3.69±1.32 | -.99 | 17.49 | .34 | -.4 |
|  | Healthy Older Adults | 22 | 4.09±.81 |  |  |  |  |
| **I understand when I need to use a Generative AI tool to seek information** |  |  |  |  |  |  |  |
|  | MCI/Dementia | 12 | 3.67±1.23 | -1.28 | 32 | .21 | -.46 |
|  | Healthy Older Adults | 22 | 4.14±.89 |  |  |  |  |
| **I find using Generative AI tools enjoyable** |  |  |  |  |  |  |  |
|  | MCI/Dementia | 13 | 3.62±1.26 | -1.59 | 33 | .12 | -.56 |
|  | Healthy Older Adults | 22 | 4.18±.85 |  |  |  |  |
| **I find Generative AI tools simple to use** |  |  |  |  |  |  |  |
|  | MCI/Dementia | 13 | 3.69±1.11 | -1.59 | 33 | .12 | -.56 |
|  | Healthy Older Adults | 22 | 4.23±.87 |  |  |  |  |
| **I can use Generative AI tools independently** |  |  |  |  |  |  |  |
|  | MCI/Dementia | 13 | 4±1.15 | -2.25 | 33 | .031 | -.79 |
|  | Healthy Older Adults | 22 | 4.68±.65 |  |  |  |  |
| **I find Generative AI tools boring** |  |  |  |  |  |  |  |
|  | MCI/Dementia | 12 | 2±1.13 | 1.11 | 17.13 | .28 | .44 |
|  | Healthy Older Adults | 22 | 1.59±.8 |  |  |  |  |
| **Using Generative AI tools is engaging** |  |  |  |  |  |  |  |
|  | MCI/Dementia | 12 | 3.58±1.16 | -.27 | 14.79 | .79 | -.11 |
|  | Healthy Older Adults | 22 | 3.68±.65 |  |  |  |  |
| **I feel overwhelmed when using Generative AI tools** |  |  |  |  |  |  |  |
|  | MCI/Dementia | 13 | 1.85±1.07 | 1.05 | 33 | .30 | .37 |
|  | Healthy Older Adults | 22 | 1.5±.86 |  |  |  |  |
|  |  |  |  |  |  |  |  |

^a^Responses from participants who reported that they use Gen-AI applications.

1=strongly disagree, 5=strongly agree.
