# Supplementary material for: Exploring Information Access in Aging Populations and Those With Dementia and Mild Cognitive Impairment in the United Kingdom: Survey and Focus Group Study
Source: JMIR Aging. 2026 Apr 21;9:e85626. doi: 10.2196/85626 (PMC13099020; doi:10.2196/85626)
Supplement: Multimedia Appendix 3 [file aging-v9-e85626-s003.docx]

Generative AI Likert response distributions (%)^a^.

| Question | Group | 1 (Strongly Disagree) | 2 | 3 | 4 | 5 (Strongly Agree) |
| --- | --- | --- | --- | --- | --- | --- |
|  |  |  |  |  |  |  |
| **“Generative AI helps me answer my questions”. To what extent do you agree with this statement?** |  |  |  |  |  |  |
|  | MCI/Dementia | 0.00% | 7.69% | 38.46% | 23.08% | 30.77% |
|  | Healthy Older Adults | 0.00% | 4.55% | 4.55% | 59.09% | 31.82% |
| **The answer Generative AI tools give me is relevant to my question** |  |  |  |  |  |  |
|  | MCI/Dementia | 0.00% | 8.33% | 0.00% | 58.33% | 33.33% |
|  | Healthy Older Adults | 0.00% | 4.55% | 4.55% | 59.09% | 31.82% |
| **I have to reword my question to get the answer I’m looking for** |  |  |  |  |  |  |
|  | MCI/Dementia | 0.00% | 16.67% | 16.67% | 58.33% | 8.33% |
|  | Healthy Older Adults | 9.09% | 18.18% | 13.64% | 45.45% | 13.64% |
| **I know how to phrase my question to a Generative AI tool** |  |  |  |  |  |  |
|  | MCI/Dementia | 7.69% | 23.08% | 30.77% | 15.38% | 23.08% |
|  | Healthy Older Adults | 0.00% | 18.18% | 4.55% | 50.00% | 27.27% |
| **Generative AI tools provide relevant answers to the question I am asking** |  |  |  |  |  |  |
|  | MCI/Dementia | 7.69% | 15.38% | 7.69% | 38.46% | 30.77% |
|  | Healthy Older Adults | 0.00% | 4.55% | 13.64% | 50.00% | 31.82% |
| **I understand when I need to use a Generative AI tool to seek information** |  |  |  |  |  |  |
|  | MCI/Dementia | 8.33% | 8.33% | 16.67% | 41.67% | 25.00% |
|  | Healthy Older Adults | 0.00% | 4.55% | 18.18% | 36.36% | 40.91% |
| **I find using Generative AI tools enjoyable** |  |  |  |  |  |  |
|  | MCI/Dementia | 7.69% | 7.69% | 30.77% | 23.08% | 30.77% |
|  | Healthy Older Adults | 0.00% | 4.55% | 13.64% | 40.91% | 40.91% |
| **I find Generative AI tools simple to use** |  |  |  |  |  |  |
|  | MCI/Dementia | 7.69% | 0.00% | 30.77% | 38.46% | 23.08% |
|  | Healthy Older Adults | 0.00% | 4.55% | 13.64% | 36.36% | 45.45% |
| **I can use Generative AI tools independently** |  |  |  |  |  |  |
|  | MCI/Dementia | 7.69% | 0.00% | 15.38% | 38.46% | 38.46% |
|  | Healthy Older Adults | 0.00% | 0.00% | 9.09% | 13.64% | 77.27% |
| **I find Generative AI tools boring** |  |  |  |  |  |  |
|  | MCI/Dementia | 50% | 8.33% | 33.33% | 8.33% | 0.00% |
|  | Healthy Older Adults | 59.09% | 22.73% | 18.18% | 0.00% | 0.00% |
| **Using Generative AI tools is engaging** |  |  |  |  |  |  |
|  | MCI/Dementia | 8.33% | 0.00% | 43.67% | 25.00% | 25.00% |
|  | Healthy Older Adults | 0.00% | 0.00% | 40.91% | 50.00% | 9.09% |
| **I feel overwhelmed when using Generative AI tools** |  |  |  |  |  |  |
|  | MCI/Dementia | 53.85% | 15.38% | 23.08% | 7.69% | 0.00% |
|  | Healthy Older Adults | 68.18% | 18.18% | 9.09% | 4.55% | 0.00% |
|  |  |  |  |  |  |  |
