# Supplementary material for: Exploring Information Access in Aging Populations and Those With Dementia and Mild Cognitive Impairment in the United Kingdom: Survey and Focus Group Study
Source: JMIR Aging. 2026 Apr 21;9:e85626. doi: 10.2196/85626 (PMC13099020; doi:10.2196/85626)
Supplement: Multimedia Appendix 4 [file aging-v9-e85626-s004.docx]

Web search Likert responses between groups^a^.

| Question | Group | n | Mean±SD | t | Df | *P* | Cohen’s d |
| --- | --- | --- | --- | --- | --- | --- | --- |
|  |  |  |  |  |  |  |  |
| **“Online search systems help me answer my questions”. To what extent do you agree with this statement?** |  |  |  |  |  |  |  |
|  | MCI/Dementia | 84 | 4.5±.67 | -1.57 | 139.26 | .118 | -.22 |
|  | Healthy Older Adults | 174 | 4.63±.55 |  |  |  |  |
| **I know how to phrase my question in an online search system** |  |  |  |  |  |  |  |
|  | MCI/Dementia | 84 | 3.9±1.03 | -2.38 | 124.9 | .019 | -.35 |
|  | Healthy Older Adults | 174 | 4.2±.73 |  |  |  |  |
| **I have to reword my question to find what I am looking for** |  |  |  |  |  |  |  |
|  | MCI/Dementia | 84 | 3.35±1.1 | 2.8 | 256 | .005 | .37 |
|  | Healthy Older Adults | 174 | 2.94±1.09 |  |  |  |  |
| **I understand when I need to use online search systems to seek information** |  |  |  |  |  |  |  |
|  | MCI/Dementia | 85 | 4.26±.68 | -2.37 | 257 | .019 | -.31 |
|  | Healthy Older Adults | 174 | 4.46±.62 |  |  |  |  |
| **I can decide if information is relevant to my question** |  |  |  |  |  |  |  |
|  | MCI/Dementia | 84 | 4.3±.74 | -2.85 | 126.8 | .005 | -.42 |
|  | Healthy Older Adults | 174 | 4.57±.54 |  |  |  |  |
| **I can find enough relevant information to answer my question** |  |  |  |  |  |  |  |
|  | MCI/Dementia | 84 | 3.9±.8 | -1.93 | 256 | .054 | -.26 |
|  | Healthy Older Adults | 174 | 4.1±.76 |  |  |  |  |
| **Information shown to me is always relevant to my question** |  |  |  |  |  |  |  |
|  | MCI/Dementia | 83 | 2.78±1 | -1.46 | 255 | .145 | -.2 |
|  | Healthy Older Adults | 174 | 2.98±.99 |  |  |  |  |
| **I can remember what I was searching for during the search process** |  |  |  |  |  |  |  |
|  | MCI/Dementia | 85 | 4±1.09 | -6.68 | 97.6 | <.001 | -1.14 |
|  | Healthy Older Adults | 173 | 4.8±.44 |  |  |  |  |
| **I feel lost during the search process** |  |  |  |  |  |  |  |
|  | MCI/Dementia | 85 | 2.39±1.2 | 5.89 | 126.2 | <.001 | .88 |
|  | Healthy Older Adults | 174 | 1.53±.85 |  |  |  |  |
| **I find the online search process engaging** |  |  |  |  |  |  |  |
|  | MCI/Dementia | 85 | 3.61±1.17 | -1.27 | 131.8 | .206 | -.19 |
|  | Healthy Older Adults | 174 | 3.79±.88 |  |  |  |  |
| **I find the online search process enjoyable** |  |  |  |  |  |  |  |
|  | MCI/Dementia | 84 | 3.58±1.06 | -1.35 | 143.6 | .179 | -.19 |
|  | Healthy Older Adults | 174 | 3.76±.9 |  |  |  |  |
| **I find online search systems simple to use** |  |  |  |  |  |  |  |
|  | MCI/Dementia | 85 | 3.78±1.08 | -3.96 | 129.2 | <.001 | -.58 |
|  | Healthy Older Adults | 174 | 4.3±.79 |  |  |  |  |
| **I can use online search systems independently** |  |  |  |  |  |  |  |
|  | MCI/Dementia | 85 | 4.32±.97 | -3 | 130.4 | .003 | -.44 |
|  | Healthy Older Adults | 174 | 4.67±.71 |  |  |  |  |
| **I find the online search process boring** |  |  |  |  |  |  |  |
|  | MCI/Dementia | 83 | 2.35±1.09 | 1.54 | 255 | .126 | .2 |
|  | Healthy Older Adults | 174 | 2.13±1.05 |  |  |  |  |
| **I feel overwhelmed when inputting my question** |  |  |  |  |  |  |  |
|  | MCI/Dementia | 85 | 2.08±1.24 | 5.02 | 118.3 | <.001 | .77 |
|  | Healthy Older Adults | 174 | 1.34±.79 |  |  |  |  |
| **I feel overwhelmed when I am presented with the results** |  |  |  |  |  |  |  |
|  | MCI/Dementia | 84 | 2.24±1.26 | 3.42 | 135.8 | <.001 | .49 |
|  | Healthy Older Adults | 174 | 1.7±1 |  |  |  |  |
| **I feel overwhelmed when deciding which information is relevant** |  |  |  |  |  |  |  |
|  | MCI/Dementia | 85 | 2.54±1.38 | 5 | 129.7 | <.001 | .74 |
|  | Healthy Older Adults | 174 | 1.7±1 |  |  |  |  |
|  |  |  |  |  |  |  |  |

^a^Responses from participants who reported that they use web search.

1=strongly disagree, 5=strongly agree.
