# Supplementary material for: Exploring Information Access in Aging Populations and Those With Dementia and Mild Cognitive Impairment in the United Kingdom: Survey and Focus Group Study
Source: JMIR Aging. 2026 Apr 21;9:e85626. doi: 10.2196/85626 (PMC13099020; doi:10.2196/85626)
Supplement: Multimedia Appendix 5 [file aging-v9-e85626-s005.docx]

Web search Likert response distributions (%)^a^.

| Question | Group | 1 (Strongly Disagree) | 2 | 3 | 4 | 5 (Strongly Agree) |
| --- | --- | --- | --- | --- | --- | --- |
|  |  |  |  |  |  |  |
| **“Online search systems help me answer my questions”. To what extent do you agree with this statement?** |  |  |  |  |  |  |
|  | MCI/Dementia | 0.00% | 1.19% | 5.95% | 34.52% | 58.33% |
|  | Healthy Older Adults | 0.00% | 0.57% | 1.72% | 31.61% | 66.09% |
| **I know how to phrase my question in an online search system** |  |  |  |  |  |  |
|  | MCI/Dementia | 3.57% | 5.95% | 17.86% | 41.67% | 30.95% |
|  | Healthy Older Adults | 0.57% | 1.72% | 9.77% | 52.87% | 35.06% |
| **I have to reword my question to find what I am looking for** |  |  |  |  |  |  |
|  | MCI/Dementia | 7.14% | 14.29% | 28.57% | 36.90% | 13.10% |
|  | Healthy Older Adults | 9.77% | 28.74% | 24.14% | 32.76% | 4.6% |
| **I understand when I need to use online search systems to seek information** |  |  |  |  |  |  |
|  | MCI/Dementia | 0.00% | 0.00% | 12.94% | 48.24% | 38.82% |
|  | Healthy Older Adults | 0.00% | 1.15% | 3.45% | 43.68% | 51.72% |
| **I can decide if information is relevant to my question** |  |  |  |  |  |  |
|  | MCI/Dementia | 1.19% | 1.19% | 5.95% | 48.81% | 42.86% |
|  | Healthy Older Adults | 0.00% | 0.00% | 2.30% | 38.51% | 59.20% |
| **I can find enough relevant information to answer my question** |  |  |  |  |  |  |
|  | MCI/Dementia | 0.00% | 3.57% | 26.19% | 46.43% | 23.81% |
|  | Healthy Older Adults | 0.00% | 2.87% | 15.52% | 50.00% | 31.61% |
| **Information shown to me is always relevant to my question** |  |  |  |  |  |  |
|  | MCI/Dementia | 8.43% | 32.53% | 26.14% | 18.07% | 4.82% |
|  | Healthy Older Adults | 5.17% | 29.89% | 31.61% | 28.74% | 4.60% |
| **I can remember what I was searching for during the search process** |  |  |  |  |  |  |
|  | MCI/Dementia | 3.53% | 8.24% | 12.94% | 35.29% | 40.00% |
|  | Healthy Older Adults | 0.00% | 0.57% | 0.57% | 14.94% | 83.91% |
| **I feel lost during the search process** |  |  |  |  |  |  |
|  | MCI/Dementia | 31.76% | 23.53% | 21.18% | 21.18% | 2.35% |
|  | Healthy Older Adults | 66.09% | 19.54% | 9.77% | 4.60% | 0.00% |
| **I find the online search process engaging** |  |  |  |  |  |  |
|  | MCI/Dementia | 5.88% | 8.24% | 34.21% | 22.35% | 29.41% |
|  | Healthy Older Adults | 1.15% | 4.02% | 31.61% | 40.80% | 22.41% |
| **I find the online search process enjoyable** |  |  |  |  |  |  |
|  | MCI/Dementia | 2.38% | 11.90% | 34.52% | 27.38% | 23.81% |
|  | Healthy Older Adults | 1.72% | 5.17% | 29.31% | 42.53% | 21.26% |
| **I find online search systems simple to use** |  |  |  |  |  |  |
|  | MCI/Dementia | 2.35% | 14.12% | 15.29% | 40.00% | 28.24% |
|  | Healthy Older Adults | 0.00% | 3.45% | 10.34% | 39.08% | 47.13% |
| **I can use online search systems independently** |  |  |  |  |  |  |
|  | MCI/Dementia | 2.35% | 4.71% | 7.06% | 30.59% | 55.29% |
|  | Healthy Older Adults | 1.15% | 1.15% | 4.02% | 16.67% | 77.01% |
| **I find the online search process boring** |  |  |  |  |  |  |
|  | MCI/Dementia | 30.12% | 18.07% | 42.17% | 6.02% | 3.61% |
|  | Healthy Older Adults | 37.93% | 20.69% | 32.76% | 7.47% | 1.15% |
| **I feel overwhelmed when inputting my question** |  |  |  |  |  |  |
|  | MCI/Dementia | 48.24% | 15.29% | 20.00% | 12.94% | 3.53% |
|  | Healthy Older Adults | 79.31% | 12.07% | 4.02% | 4.02% | 0.57% |
| **I feel overwhelmed when I am presented with the results** |  |  |  |  |  |  |
|  | MCI/Dementia | 41.67% | 17.86% | 17.86% | 20.24% | 2.38% |
|  | Healthy Older Adults | 59.77% | 18.97% | 13.79% | 6.32% | 1.15% |
| **I feel overwhelmed when deciding which information is relevant** |  |  |  |  |  |  |
|  | MCI/Dementia | 34.12% | 15.29% | 22.35% | 18.82% | 9.41% |
|  | Healthy Older Adults | 59.20% | 21.26% | 10.29% | 7.47% | 1.15% |
|  |  |  |  |  |  |  |
